# A Comparison of the Effects of Empagliflozin and Sitagliptin, When Combined With Metformin, on Lipid Levels in Patients with Type 2 Diabetes: A Clinical Investigation

**DOI:** 10.7759/cureus.44709

**Published:** 2023-09-05

**Authors:** Mazhar Ahmed, Asjad Saeed, Muhammad Zarar Khan, Sana Z Javaid, Farhan Aslam, Savida Ilyas Dar

**Affiliations:** 1 Internal Medicine, Federal Government Polyclinic Hospital, Islamabad, PAK; 2 Internal Medicine, Shifa College of Medicine, Shifa International Hospital, Islamabad, PAK

**Keywords:** sitagliptin, metformin, lipid profile, empagliflozin, diabetes mellitus

## Abstract

Introduction

Type 2 diabetes (T2D) is emerging as a major global health concern. An associated condition, dyslipidemia, which acts as a significant modifiable risk factor for T2D, exhibits variations across different ethnicities and socioeconomic backgrounds. While many patients rely on metformin as their primary treatment, it does not always effectively control hyperglycemia. As a result, there is a growing need for adjunctive treatments, including sodium-glucose cotransporter-2 (SGLT2) and dipeptidyl peptidase-4 (DPP-4) inhibitors. This study evaluated the comparative effects of empagliflozin (an SGLT2 inhibitor) and sitagliptin (a DPP-4 inhibitor), both combined with metformin, on the lipid profiles of individuals with T2D.

Methods

Over six months at the Federal Government Polyclinic Hospital in Islamabad, we enrolled 126 participants diagnosed with T2D. Using a nonprobability consecutive sampling technique, we divided them into two groups. Group A received metformin and empagliflozin, while Group B was administered metformin and sitagliptin. We assessed their fasting lipid profiles three months into the treatment.

Results

Both groups consisted of 63 patients each. We observed that those in Group B, treated with sitagliptin and metformin, demonstrated a more significant reduction in total cholesterol and low-density lipoprotein-C levels than those in Group A, treated with empagliflozin and metformin. This difference proved to be statistically meaningful.

Conclusion

The combination of sitagliptin and metformin showed enhanced benefits in lipid profile management compared to the combination of empagliflozin and metformin in patients with T2D. This discovery underscores the need for holistic treatment modalities that factor in blood glucose levels and cardiovascular health.

## Introduction

Type 2 diabetes (T2D) is an increasing public health concern worldwide. Estimates predict a doubling of T2D cases globally in the next 25 years [1]. In Pakistan, the T2D incidence is approximately 11%, projected to rise to 15% of the population by 2030. The primary treatment involves lifestyle modifications and metformin as the first-line treatment [2].

Dyslipidemia, characterized by high total cholesterol, increased triglycerides, low high-density lipoprotein (HDL), and elevated low-density lipoprotein cholesterol (LDL-C) levels, is a significant modifiable risk factor for T2D. Though common in T2D patients, variations exist across ethnicities and socioeconomic conditions, reflecting differences in healthcare access [3-6]. Insulin plays a crucial role in fatty acid metabolism, and factors such as diabetes, low insulin levels, and insulin resistance can influence lipid compositions [7,8].

While lifestyle changes accompanied by metformin serve as first-line therapy for T2D, many patients remain hyperglycemic with this regimen, necessitating second-line medications [9]. Sodium-glucose cotransporter-2 (SGLT2) inhibitors represent a novel drug class under investigation. These drugs lower blood glucose by reducing renal glucose reabsorption without affecting insulin secretion [10,11]. Empagliflozin, an oral SGLT2 inhibitor, has demonstrated efficacy both as standalone and in combination with other oral anti-hyperglycemia (OAH) drugs in several randomized controlled trials [12]. Dipeptidyl peptidase-4 (DPP-4) inhibitors like sitagliptin are vital second-line or monotherapy anti-hyperglycemia drugs, decreasing plasma DPP-4 activity by 80% and elevating active glucagon-like peptide-1 levels, thus enhancing cell functionality [13,14].

Research indicates that sitagliptin can effectively decrease total cholesterol, LDL-C, and non-HDL-C in T2D patients with elevated baseline triglyceride levels [15,16]. This study aimed to compare the lipid profile effects of empagliflozin and sitagliptin combined with metformin in T2D patients.

## Materials and methods

We conducted this study at the Federal Government Polyclinic Hospital, Islamabad, over six months, securing approval from the Shaheed Zulfiqar Ali Bhutto Medical University ethical review board (approval no. F.20-4/AS&RB-M/2020/SZABMU). All participants provided informed consent.

Eligible participants were diagnosed with T2D, were aged 18-60 years, and were on metformin for over three months with recent glycated hemoglobin (HbA1c) levels exceeding 7%. Using a lottery system, we employed a nonprobability consecutive sampling model and divided 126 patients equally into two groups (Groups A and B). Several exclusion criteria were set, including HbA1c levels above 10%, concurrent anti-obesity medication, known thyroid disorders, chronic liver or renal diseases, pregnancy, lactation, drug misuse, poor medication adherence, and missing data.

Group A patients received 1000 mg metformin twice daily and 10 mg empagliflozin once daily. In contrast, Group B patients were administered 1000 mg metformin twice daily and 50 mg sitagliptin twice daily. We monitored all participants after three months, with the hospital laboratory evaluating fasting lipid profiles. Demographic data and study parameters were collected using a standard proforma. For data analysis, we utilized IBM SPSS Statistics for Windows, version 23.0 (IBM Corp., Armonk, NY), with significance set at p<0.05.

## Results

Of the 126 trial participants, Group A had an average age of 55.2 ± 6.2 years, and Group B’s average age was 51.8 ± 6.3 years. Diabetes persisted for a mean of 4.3 ± 1.3 years in Group A and 4.3 ± 1.4 years in Group B. Group A consisted of 43 men (68.3%) and 20 women (31.7%), and Group B had 60 men (95.2%) and 3 women (4.8%). Table 1 illustrates fasting lipid profile changes between baseline and the three-month mark. As demonstrated, Group B (sitagliptin + metformin) exhibited a more pronounced improvement in total cholesterol and LDL-C levels than Group A (empagliflozin + metformin), with this difference being statistically significant.

**Table 1:** Lipid profiles for study groups A and B LDL-C, low-density lipoprotein cholesterol; HDL-C, high-density lipoprotein cholesterol; SD, standard deviation *p<0.05 is considered statistically significant.

Lipid profile	Groups (n=63 each)	Mean	SD	p-value
Total cholesterol at baseline (mg/dL)	Group A (empagliflozin + metformin)	191.87	16.01	0.190
	Group B (sitagliptin+ metformin)	195.38	13.77	
Total cholesterol af­ter 3 months (mg/dL)	Group A (empagliflozin + metformin)	190.16	15.61	0.455
	Group B (sitagliptin + metformin)	192.03	12.27	
Total cholesterol change from baseline (mg/dL)	Group A (empagliflozin + metformin)	-1.71	3.29	0.004*
	Group B (sitagliptin + metformin)	-3.35	2.88	
LDL-C at baseline (mg/dL)	Group A (empagliflozin + metformin)	156.89	12.99	0.001*
	Group B (sitagliptin + metformin)	164.84	12.05	
LDL-C after 3 months (mg/dL)	Group A (empagliflozin + metformin)	155.37	13.01	0.009*
	Group B (sitagliptin + metformin)	161.05	10.80	
LDL-C change from baseline (mg/dL)	Group A (empagliflozin + metformin)	-1.52	2.40	<0.001*
	Group B (sitagliptin + metformin)	-3.79	3.62	
HDL-C at baseline (mg/dL)	Group A (empagliflozin + metformin)	39.97	1.51	0.593
	Group B (sitagliptin + metformin)	40.10	1.12	
HDL-C after 3 months (mg/dL)	Group A (empagliflozin + metformin)	39.56	1.41	0.015*
	Group B (sitagliptin + metformin)	40.11	1.11	
HDL-C change from baseline (mg/dL)	Group A (empagliflozin + metformin)	-0.41	0.99	0.003*
	Group B (sitagliptin + metformin)	0.02	0.52	
Triglycerides at baseline (mg/dL)	Group A (empagliflozin + metformin)	190.67	21.08	0.070
	Group B (sitagliptin + metformin)	184.67	15.29	
Triglycerides after 3 months (mg/dL)	Group A (empagliflozin + metformin)	188.95	20.80	0.033*
	Group B (sitagliptin + metformin)	181.97	15.01	
Triglyceride change from baseline (mg/dL)	Group A (empagliflozin + metformin)	-1.71	3.28	0.062
	Group B (sitagliptin + metformin)	-2.70	2.54	

## Discussion

Our study revealed that in patients with T2D, the combination of sitagliptin and metformin (Group B) enhanced the overall lipid profile more effectively than the combination of empagliflozin and metformin (Group A), particularly in terms of total cholesterol and LDL-C.

A previous research indicated that empagliflozin did not significantly alter lipid parameters from baseline in patients with T2D [17]. While another study observed a reduction in total cholesterol and LDL-C when empagliflozin was combined with either insulin or OAH medications, these changes lacked statistical significance [18]. The participants’ demographic characteristics and baseline variables in these prior studies align closely with ours. Contrarily, our investigation observed a statistically significant difference in the reduction of total cholesterol and LDL-C levels between the two groups, potentially attributed to the combination with metformin.

Several studies have reported significant improvements in total cholesterol and triglyceride levels with sitagliptin [15,16]. These studies compared sitagliptin’s efficacy against other diabetes medications, such as sulfonylureas combined with metformin and pioglitazone combined with either metformin or sulfonylureas. We examined the differential effects of empagliflozin and sitagliptin on lipid parameters in T2D, juxtaposing an SGLT-2 inhibitor against a DPP-4 inhibitor. Nevertheless, our findings are in harmony with these prior studies.

T2D, a multifaceted metabolic disorder, leads to various complications. Hence, therapeutic approaches should target glycemic control and aim to mitigate associated complications. While empagliflozin has demonstrated notable advantages concerning glycemic control and weight loss [19,20], our study did not observe significant enhancements in lipid parameters in the empagliflozin and metformin group (Group A).

This study also had several limitations. The limited sample size and a short follow-up duration of only three months may restrict the generalizability of our findings. Without long-term data, it remains uncertain how consistent and enduring these observed effects may be. The study design did not permit blinding participants to their assigned treatment groups, introducing the potential for bias in reporting outcomes. Additionally, there was no stringent control over confounding variables that might have influenced the lipid parameters in T2D patients, potentially skewing results. A more comprehensive approach that considers various associated factors and ensures tighter controls would be essential in future research.

## Conclusions

Our study aimed to compare the effects of empagliflozin versus sitagliptin, both combined with metformin, on the lipid profiles of patients with T2D. Our most salient findings revealed that the combination of sitagliptin and metformin was more effective in reducing total cholesterol and LDL-C levels than empagliflozin combined with metformin. The study illuminates the significance of considering lipid parameter alterations when selecting adjunct treatments for T2D. From a broader medical perspective, these findings underscore the necessity of a holistic approach to diabetic treatment, considering both glycemic control and cardiovascular risk factors. When optimizing treatment regimens for T2D patients, clinicians should aim for glucose regulation and improved lipid parameters, potentially reducing associated cardiovascular risks.
